# *Corynebacterium glutamicum* promoters: a practical approach

**DOI:** 10.1111/1751-7915.12019

**Published:** 2013-01-10

**Authors:** Miroslav Pátek, Jiří Holátko, Tobias Busche, Jörn Kalinowski, Jan Nešvera

**Affiliations:** 1 Institute of Microbiology AS CR, v. v. i.CZ-14220, Prague 4, Czech Republic; 2 Centrum für Biotechnologie, Universität Bielefeld33594, Bielefeld, Germany

## Abstract

Transcription initiation is the key step in gene expression in bacteria, and it is therefore studied for both theoretical and practical reasons. Promoters, the traffic lights of transcription initiation, are used as construction elements in biotechnological efforts to coordinate ‘green waves’ in the metabolic pathways leading to the desired metabolites. Detailed analyses of *Corynebacterium glutamicum* promoters have already provided large amounts of data on their structures, regulatory mechanisms and practical capabilities in metabolic engineering. In this minireview the main aspects of promoter studies, the methods developed for their analysis and their practical use in *C. glutamicum* are discussed. These include definitions of the consensus sequences of the distinct promoter classes, promoter localization and characterization, activity measurements, the functions of transcriptional regulators and examples of practical uses of constitutive, inducible and modified promoters in biotechnology. The implications of the introduction of novel techniques, such as *in vitro* transcription and RNA sequencing, to *C. glutamicum* promoter studies are outlined.

## Introduction

*Corynebacterium glutamicum* is known as an industrial microorganism, in particular due to its use in the large-scale production of various amino acids (e.g. glutamate and lysine), which began as early as in the 1950s. Because this Gram-positive bacterium is generally recognized as a safe organism suitable for biotechnological processes, its use in the production of vitamins (Hüser *et al*., 2005), oligonucleotides (Vertes *et al*., 2012), organic acids (Wieschalka *et al*., 2012), higher alcohols (Blombach and Eikmanns, 2011), diamines (Kind *et al*., 2010) and polymers (Song *et al*., 2012) has widened its utility. In the initial period of strain development, mainly mutagenesis resulting in alterations in gene repression and/or enzymes feedback inhibition was applied. The later developed procedures of genetic engineering contributed to strain improvement by constructing strains with an increased or decreased expression of genes involved in the respective metabolic pathways and connected metabolic branches. These approaches mainly consist of amplifying the genes involved in the biosynthesis pathways and/or their overexpression using strong promoters. The determination of the complete *C. glutamicum* genome sequence, the establishment of techniques for the global analysis of gene expression and cell metabolism (transcriptomics, proteomics, metabolomics, fluxomics) (Wendisch *et al*., 2006) and a deeper knowledge of the regulatory network of *C. glutamicum* metabolism (Baumbach *et al*., 2009; Schröder and Tauch, 2010) have enabled the use of more sophisticated modifications of cell capabilities. Combining the genome-wide techniques and targeted modifications of gene expression, particularly the use of various promoters, have already resulted in the construction of biotechnologically promising strains (Becker and Wittmann, 2012; Vertes *et al*., 2012).

A **promoter** in the strict sense (core promoter) is a DNA sequence (approximately 40–50 bp) that specifies the binding site for an RNA polymerase (RNAP) holoenzyme and the transcriptional start point (TSP). Precisely localizing the TSP and the corresponding key promoter sequence motifs as well as characterizing the promoter activity under various conditions provide a basis for the rational use of the promoter for biotechnological purposes. The promoter activity and conditions under which the promoter is active may be markedly affected by the flanking sequences covering as much as hundreds of nucleotides. This **promoter region** may include binding sites for transcriptional regulators (TRs) and other regulatory elements. The remarkable progress made in the experimental characterization of TRs in *C. glutamicum* in recent years (reviewed by Schröder and Tauch, 2010) has provided comprehensive knowledge of the regulatory network of transcription in *C. glutamicum*.

The replacement of native promoters with stronger or weaker ones (constitutive or inducible) or mutagenesis of specific nucleotides within the promoter sequences are the main methods used for the directed modulation of gene expression in bacteria. Tightly regulated inducible promoters are usually used if the increased synthesis of the gene product has a detrimental effect on cell viability. Constitutive promoters can be used to increase the expression of genes involved in one metabolic pathway which results in an increased metabolic flux to the desired final metabolite. The modified promoters constructed by site-directed mutagenesis of the specific nucleotides within the promoter sequences can be used to fine tune gene expression.

This minireview covers many aspects of promoter studies, including the ways they are detected and their applications in biotechnology.

## Housekeeping and stress-induced *C. glutamicum* promoters

### Promoter classes

Promoters can be classified according to the σ subunits (factors) of the RNAP holoenzymes, which are responsible for the recognition of the respective promoter sequences. The *C. glutamicum* genome codes for seven different sigma factors (all of them of the σ^70^-type) falling into three groups according to the classification by Gruber and Gross (2003): the primary sigma factor σ^A^ (Group 1), the primary-like sigma factor σ^B^ (Group 2) and alternative factors σ^C^, σ^D^, σ^E^, σ^H^ and σ^M^, which are all members of Group 4, also called extracytoplasmic function (ECF) σ factors (Pátek and Nešvera, 2011). *C. glutamicum* promoters are bound by a holo-RNAP formed by the subunits α_2_ββ′ω + σ. The promoters of housekeeping genes (recognized by σ^A^) form the largest described group. Their consensus sequence is defined, although their key specific sequence motifs (−35 and −10 sequences) exhibit a wide variability (low level of conservation). Most *C. glutamicum* promoters controlled by alternative σ factors are considered to be stress-induced. The characteristics of these promoters are still being discovered, although σ^B^-, σ^H^-and σ^M^-dependent promoters have already been analysed to some extent.

### Promoters of housekeeping genes

It is assumed that the majority of the genes which are essential for the rapid growth of bacteria in minimal medium are transcribed from the promoters recognized by RNAP containing the primary sigma factor. The promoters of these housekeeping genes and their mutant derivatives are the transcriptional elements most frequently used for the modulation of bacterial gene expression in biotechnological applications. The key features of the promoters of housekeeping genes have been described in detail in *Escherichia coli*. The consensus sequences of these key promoter motifs in *E. coli* (TTGACA in the −35 region and TATAAT in the −10 region) have been defined. Two other elements that were only detected in some promoters are the extended −10 element TG (**TG**NTATAAT) and the UP element (an approximately 20 nt AT-rich sequence) located just upstream of the −35 motif (Browning and Busby, 2004).

The basic structure of *C. glutamicum* housekeeping promoters (−35 and −10 motif) is similar to the consensus sequences of *E. coli* and other eubacterial promoters recognized by primary sigma factors. The statistical consensus sequence of these key motifs in the 159 *C. glutamicum* promoters assumed to be σ^A^-dependent has been derived (Pátek and Nešvera, 2011). It consists of the −35 region TTGNCA and the extended −10 region GN**TA**NAN**T**NG (nt in bold are found in more than 80% of the sequences, the other nt appear in more than 35% of sequences; core hexamers are underlined). The nucleotides in the *C. glutamicum* −35 consensus are much less conserved than those in the *E. coli* consensus and cannot be identified in many *C. glutamicum* promoters.

In biotechnology, particularly strong promoters are preferred for the construction of expression systems. The statistical consensus of housekeeping promoters does not necessarily represent the strongest promoter, since the structure of promoters and their strength evolved to increase the fitness of the cell rather than to achieve the highest gene expression. Similarly to *E. coli*, additional elements of *C. glutamicum* promoter sequences that are not conserved may affect the promoter's activity. Mutational studies of *C. glutamicum* promoters showed that the presence of the TG dimer within the extended −10 region increases promoter strength (Vašicová *et al*., 1999; Hänssler *et al*., 2009; Holátko *et al*., 2009). We can therefore consider the extended −10 motif TGnTATAATnG (Vašicová *et al*., 1999) and −35 motif TTG^A^/_C_CA (Asakura *et al*., 2007) (core hexamers are underlined) to be a functional consensus which defines the sequences of the strongest core promoter elements. However, even combining these two optimal elements does not guarantee the highest transcription efficiency in *C. glutamicum*.

It was believed that the primary factor σ^A^ is responsible for the transcription of all housekeeping genes. Recently, genes encoding the enzymes of glucose catabolism were found to be expressed from σ^B^-controlled promoters during the exponential growth phase of *C. glutamicum* cultures (Ehira *et al*., 2008). This finding led to the conclusion that some proportion of housekeeping genes may be transcribed from σ^B^-dependent promoters (Ehira *et al*., 2008).

### Stress-induced promoters

The expression of many *C. glutamicum* genes was found to be controlled by alternative sigma factors σ^B^, σ^H^ or σ^M^ (Larisch *et al*., 2007; Nakunst *et al*., 2007; Ehira *et al*., 2008; 2009; Busche *et al*., 2012). Most of the *C. glutamicum* genes controlled by these sigma factors are involved in the cell adaptation to limited growth during the onset of the stationary phase and in the responses to various stresses, e.g. heat or cold shock and oxidative and cell surface stress. A number of the promoters, which are thought to be recognized by a holo-RNAP containing σ^B^, σ^H^ or σ^M^ were characterized.

SigB-dependent genes, which are mostly expressed in the transition phase between the exponential and stationary growth phases, are responsible for stress-defence functions, transport, amino acid metabolism and regulatory processes (Larisch *et al*., 2007; Ehira *et al*., 2008). In addition to the stress-induced genes, the transcription of genes involved in glucose consumption was proven to be σ^B^-controlled both under conditions of oxygen deprivation and during rapid aerobic growth (Ehira *et al*., 2008). Based on 13 localized σ^B^-specific promoters it was found that the σ^B^-specific promoters contain −10 sequences which are very similar to the −10 motif of the σ^A^-specific promoters. Potential differences between the σ^A^ and σ^B^-specific promoters might be recognized by the mutagenesis of particular nucleotide positions and by *in vitro* transcription experiments. However, the first *C. glutamicum* σ^A^-dependent promoter proved by *in vitro* transcription (P*per*; Holátko *et al*., 2012) was also recognized by RNAP + σ^B^ (J. Holátko and M. Pátek, unpublished). This suggests that at least some σ^A^-dependent promoters might be also recognized by σ^B^ under specific physiological conditions *in vivo*. *C. glutamicum* σ^B^ can thus be considered to be not only a backup σ factor for unfavourable conditions (Larisch *et al*., 2007) but also another σ factor that recognizes some housekeeping promoters during rapid growth (Ehira *et al*., 2008). The σ^B^-dependent promoters may therefore be used to enhance the expression of the selected genes under various stress conditions or during the limited growth of *C. glutamicum* strains constructed for use in biotechnological processes.

SigH-dependent promoters form the largest group of currently described stress-induced *C. glutamicum* promoters. The *C. glutamicum* genes controlled by σ^H^ were mainly discovered by differential expression microarrays using the WT strain, *sigH*-overexpressing strain and strains with a deletion within the *sigH* gene or *rshA* gene encoding the specific anti-σ^H^ protein RshA (Ehira *et al*., 2009; Busche *et al*., 2012). Most of the σ^H^-regulated genes are involved in the heat shock response. In total, 27 σ^H^-specific promoters were localized by experimental mapping of the respective TSPs (for an overview, see Busche *et al*., 2012). In addition, further 18 potential σ^H^-specific promoters were predicted using bioinformatic analysis (Busche *et al*., 2012). The resulting set of 45 sequences of assumed σ^H^-specific promoters enabled their consensus sequence to be reliably derived. The core of the consensus is formed by the highly conserved sequence **GGAA** – N_18–21_ – **GTT** (with the exception of the second A found in 88% of the promoters, all these nt are found in 97% to 100% of the promoters), which was also described in the promoters of stress-response genes in mycobacteria (Rodrigue *et al*., 2006) and in *Streptomyces* (Paget *et al*., 1998). Other nucleotides in the extended form of the consensus sequence of *C. glutamicum* σ^H^-specific promoters, (−35) ^G^/_T_**GGAA**^T^/_C_^A^/_T_ and (−10) ^C^/_T_**GTT**^G^/_A_^A^/_T_^A^/_T_ are much less conserved (in more than 40% of promoters). The σ^H^-dependent promoters have not yet been utilized in constructions of biotechnologically important *C. glutamicum* strains; however, their potential use in heat-induced gene expression is envisaged.

There are only four promoters which were described as σ^M^-dependent (Nakunst *et al*., 2007). The same genes (involved in the oxidative stress response) and their promoters were, however, defined as σ^H^-dependent in another study (Ehira *et al*., 2009). Since the P*sigM* promoter is most likely recognized by RNAP + σ^H^ (Nakunst *et al*., 2007), the dependence of these genes on σ^H^ may be indirect. We intend to address this issue in the future by using the novel *in vitro* transcription system for *C. glutamicum*.

## Localization and characterization of *C. glutamicum* promoters

### Transcriptional start determination

**Figure 1:**
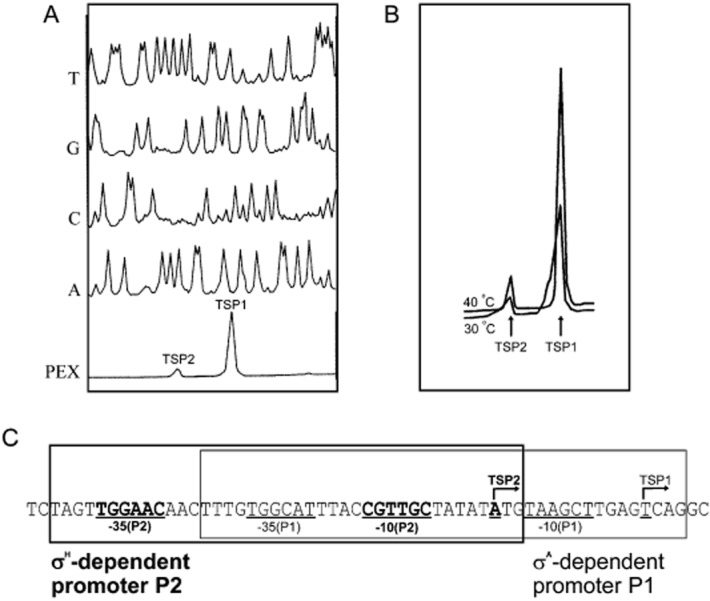
Mapping of TSPs by primer extension (PEX) and deducing respective promoters. Analysis of *C. glutamicum* *dnaK* gene transcription inducible by heat shock is shown (Barreiro *et al*., 2004).A. Determination of two *dnaK* TSPs by non-radioactive PEX technique. The bottom peaks (PEX) represent cDNA synthesized by reverse transcription using RNA from *C. glutamicum*. The peaks generated by the automatic sequencer (T, G, C, A) represent the products of sequencing reactions run with the same fluorescein-labelled primer as that used for PEX. The TSP nucleotides were determined by comparing the positions of the primer extension products and the sequencing signal.B. Quantitative comparison of signals representing reverse *dnaK* transcripts synthesized on RNA templates isolated from cells cultivated under various conditions (30°C or 40°C).C. Defining two *dnaK* promoters. The nucleotides of TSPs and the core promoter sequences are underlined, the sequences representing the σ^H^-dependent promoter (P2) are shown in bold and the sequences of the σ^A^-dependent promoter (P1) are in plain font. The boxes represent the approximate regions of the P1 promoter (thin line) and the P2 promoter (thick line).

A number of TSPs pertinent to *C. glutamicum* promoters were determined by radioactive (Pátek *et al*., 1996; Möker *et al*., 2004; Suda *et al*., 2008) or non-radioactive (Pátek *et al*., 2003a; Barreiro *et al*., 2004; Holátko *et al*., 2009) primer extension analysis (PEX). Using PEX, several TSPs of a gene could be discovered in a single experiment (Pátek *et al*., 2003a; Barreiro *et al*., 2004; Busche *et al*., 2012). The specific transcripts defined by TSPs can be indirectly quantified by integrating the electropherogram signals produced by PEX (Fig. 1) (Barreiro *et al*., 2004). The 5′RACE technique has also been widely used for TSP mapping (Nolden *et al*., 2001; Larisch *et al*., 2007; Nakunst *et al*., 2007; Ehira *et al*., 2009; Schröder *et al*., 2010). Recently, the use of RNA sequencing for *C. glutamicum* resulted in a breakthrough in the scale and accuracy of TSP determination (see *RNA sequencing*).

### Promoter activity assessment

Reporter systems are convenient tools for screening promoters and measuring promoter activities. The commonly used reporter genes in *C. glutamicum* encode chloramphenicol acetyl transferase (*cat*), β-galactosidase (*lacZ*), aminoglycoside phosphotransferase (*aph*), α-amylase (*amy*) and green fluorescent protein (*gfp*) (for a review, see Pátek and Nešvera, 2012). The promoter probe vectors pET2 (*cat*; Vašicová *et al*., 1998) and pEPR1 (*gfp*; Knoppová *et al*., 2007) are most frequently used for promoter analyses. Their use in analyses of promoter strength and in elucidating the regulatory mechanisms of transcription have recently been summarized (Nešvera and Pátek, 2011).

To test the expression from a promoter controlled by a single copy of a TR gene in the cell, the integrative promoter-test vectors pRIM2 (*cat*; Vašicová *et al*., 1998) and pCRA741 (*lacZ*; Inui *et al*., 2007) can be used.

### Predictions of promoters by bioinformatic tools

Many systems of promoter detection using bioinformatic analyses have been developed. Computer-assisted searches for promoter sequences in ten bacterial species (including *C. glutamicum*) using a genomic distribution of hexanucleotide pairs within intergenic regions has been described as a promising general tool for the prediction of promoters (P.E. Jacques *et al*., 2006). However, such analyses based only on the presence of putative −10 and −35 hexamers generate many false positives in their promoter predictions. The web-based tool BioProspector (Liu *et al*., 2001) has been used to detect the sequence motifs of *C. glutamicum* σ^H^-specific promoters (Busche *et al*., 2012).

**Figure 2:**
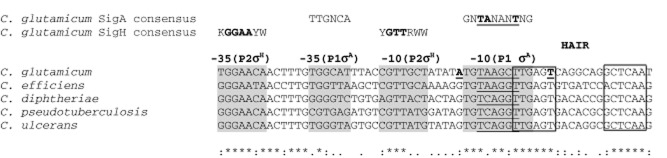
Alignment of *dnaK* promoter regions of five species of genus *Corynebacterium*. The conserved putative −10 and −35 sequences of σ^A^-dependent promoter P1 and σ^H^-dependent promoter P2 are shaded. The −10 core hexamer of P1 is underlined. The experimentally determined TSPs pertinent to the *C. glutamicum* P1 (T) and P2 (A) promoters (Barreiro *et al*., 2004) are in bold and underlined. The boxes represent the conserved HAIR sequences (binding sites for the HspR repressor). The positions with identical nucleotides in all sequences are indicated with an asterisk (*); positions with identical nucleotides in 4/5 and 3/5 sequences are indicated with a colon (:) and a dot (.) respectively. The consensus sequences of *C. glutamicum* σ^A^-and σ^H^-dependent promoters are shown in the IUPAC code (K = G or T; Y = C or T; R = A or G; W = A or T; N = A,G,T,C) above the alignment (Pátek and Nešvera, 2011; Busche *et al*., 2012).

A relatively simple sequence analysis aimed at predicting promoter sequences in *C. glutamicum* includes aligning the promoter regions of orthologous genes from related corynebacterial species. By using multiple alignments of promoter regions coming from related genomes, the promoter sequence motifs can be recognized. In addition to promoter sequences, other highly conserved regions can be identified, such as binding sites for TRs. An example of such an analysis of the *dnaK* promoter regions of five species of the genus *Corynebacterium* is shown in Fig. 2.

### RNA sequencing

**Figure 3:**
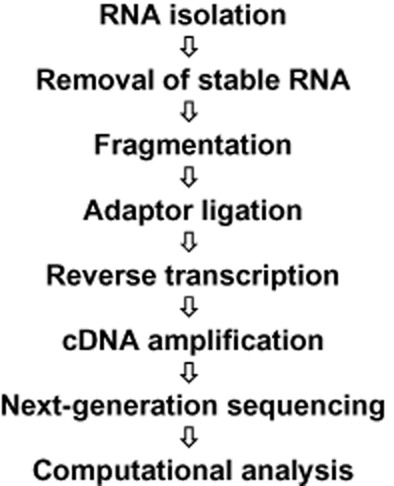
General workflow for transcriptome sequencing (RNA-seq) in bacteria.

High-throughput sequencing technologies of whole transcriptomes (RNA-seq) have recently provided new possibilities for analysing transcripts in a genome-wide manner (van Vliet, 2010). RNA-seq enables the determination of an enormous number of TSPs and consequently the localization of the respective promoters. The construction of a genome-wide promoterome thus represents a qualitative as well as a quantitative step forward in promoter studies, since it gives a single nucleotide resolution of TSPs (+1) as well as a relative number of mRNAs with intact (primary) 5′-ends. Starting with isolated total RNA (Fig. 3) the first step is to remove the large amounts of stable RNA species (rRNAs and tRNAs) which make up more than 95% of the transcripts in bacteria. A specific requirement in sequencing is the appropriate length of the cDNA fragment to be sequenced, thus influencing the fragmentation that is carried out by shearing or metal-catalysed hydrolysis. A general requirement is to keep track of which strand of the genome the RNA was transcribed from. This information is preserved by using specific adaptor sequences that are directly ligated to the RNA. The 3′-adaptor is used for first-strand cDNA (ss-cDNA) synthesis by reverse transcription with a specific primer. Both adaptors then serve to amplify all ss-cDNAs to ds-cDNAs by a low-cycle PCR. The resulting material, ds-cDNA fragments in the desired size range, can then be used in Next-Generation Sequencing. In an ideal case, where sensitivity and dynamic range are focused on, sequencing might result in several million DNA sequences (reads) that need to be assigned to the genome by mapping algorithms, and mapping data that need to be converted to definitions of transcripts, their 5′-and 3′-ends as well as to quantitative information.

**Figure 4:**
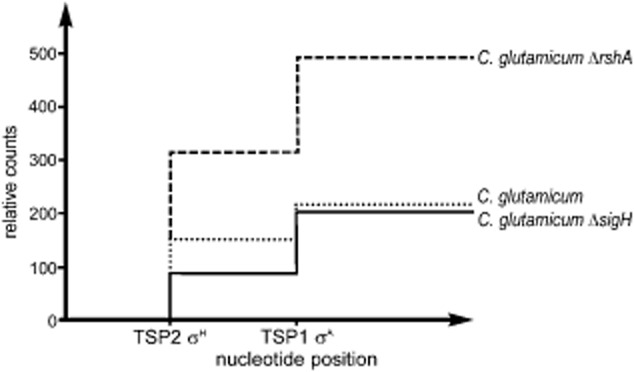
Determination of TSPs of the *C. glutamicum* *dnaK* gene by transcriptome sequencing. The reads (sequences) derived from RNA-seq experiments that map the 5′-ends of the transcripts driven from the σ^A^-and the σ^H^-specific promoters are shown. The *y* axis denotes relative transcription efficiency as the number of reads normalized to the total number of reads in the respective RNA-seq experiment. The *C. glutamicum* Δ*sigH* mutant and the *C. glutamicum* Δ*rshA* mutant (with inactivated anti-sigma factor RshA) exhibit a different activity of the σ^H^-specific P2*dnaK* promoter, whereas the activity of the σ^A^-driven P1*dnaK* promoter remained unaffected in the Δ*sigH* strain (T. Busche and J. Kalinowski, unpublished).

We applied the RNA-seq technique to studies of the *C. glutamicum* transcriptome and present here an example of its use in the analysis of *dnaK* promoters P1 and P2 (T. Busche and J. Kalinowski, unpublished; Fig. 4) In a novel modification of RNA-seq called ‘differential RNA-seq’ (Sharma *et al*., 2010) a terminator exonuclease that degrades processed transcripts (with 5′P) is used to leave nothing but transcripts with a native 5′-end (5′PPP). This helps to unequivocally identify TSPs and promoter sequences. In the application example shown in Fig. 4, we used two different *C. glutamicum* mutant strains, lacking σ^H^ and RshA (anti-σ^H^) respectively. This technique is not only comprehensive, but able to zoom in on genes of interest, displaying TSPs recognized by different sigma factors as well as their quantitative discrimination. The transcription of the *dnaK* gene (Barreiro *et al*., 2004; 2005) is driven by two overlapping promoters of different classes. The P1 promoter is recognized by the housekeeping sigma factor σ^A^ and the P2 promoter by σ^H^ (Fig. 1). The deletion of the *sigH* gene led to a substantial reduction in the number of reads representing the specific 5′-end of the σ^H^-dependent transcript, whereas the deletion within the *rshA* gene, encoding the σ^H^ anti-sigma factor RshA, led to an increase in the number of reads. The σ^A^-dependent TSP was largely unaffected. These results clearly confirm that the P2*dnaK* promoter is controlled by σ^H^. In addition to the quantitative information and single-nucleotide resolution of TSPs provided by this technique, the data support the notion that the P2*dnaK* promoter is also recognized by at least one other sigma factor, responsible for the observed promoter activity in the *sigH* deletion mutant. According to the results of *in vitro* transcription, it is σ^E^ which recognizes the P2*dnaK* promoter, in addition to σ^H^ (J. Holátko and M. Pátek, unpublished). This conclusion documents the usefulness of combining different experimental approaches to the analysis of promoters.

### *In vitro* transcription

An *in vitro* transcription assay generally provides a powerful tool to study transcriptional regulation in bacteria. An *in vitro* transcription system, which uses purified components of the transcription machinery, mimics many features of *in vivo* transcription and thus forms a basis for the detailed analyses of transcription initiation, elongation and termination. It complements other techniques analysing promoter – RNAP interactions, the functions of TRs and promoters of different classes. *In vitro* transcription has been broadly used to characterize the properties of RNAP from *E. coli* (Ross and Gourse, 2009) and *Bacillus subtilis* (Fujita, 1999). The *in vitro* transcription system also enabled promoters of various classes to be associated with particular sigma subunits of RNAP in the human pathogen *Mycobacterium tuberculosis* (J.F. Jacques *et al*., 2006) that is taxonomically related to corynebacteria. We have recently developed an *in vitro* transcription system for *C. glutamicum* (Holátko *et al*., 2012). This system consists of a *C. glutamicum* RNA polymerase core (α2, β, β′), a sigma factor and a promoter-carrying DNA template that is specifically recognized by the RNAP holoenzyme formed. The RNAP core was purified from the *C. glutamicum* strain with a modified *rpoC* gene, which produced a His-tagged β′ subunit. The *C. glutamicum sigA*, *sigB*, *sigE* and *sigH* genes were cloned and overexpressed using an *E. coli* plasmid vector and the respective σ subunits were purified by affinity chromatography. In the assays containing promoter DNA templates and the reconstituted *C. glutamicum* holo-RNAPs, specific transcripts were formed in all cases, confirming the functionality of the *in vitro* transcription system and its usefulness in determining sigma specificity in recognizing particular promoters (Holátko *et al*., 2012). This *C. glutamicum in vitro* transcription system is a novel tool that can be used to identify all classes of promoters (i.e. recognized by any of seven sigma factors of *C. glutamicum*) and to analyse transcriptional control by various regulatory proteins in *C. glutamicum*. We have already proved that some *C. glutamicum* promoters are *in vitro* recognized by two sigma factors. The P2 promoter of *dnaK* (encoding a heat shock protein) and the promoter of *sigB* (encoding the sigma factor σ^B^) were found to be recognized by both RNAP + σ^H^ and RNAP + σ^E^ (J. Holátko, unpublished). This is in agreement with the results of experiments in which *dnaK* and *sigB* transcription was induced in response to heat shock and in response to cell surface stress controlled by σ^H^ and σ^E^ respectively.

## Transcriptional regulators

The TRs (activators and repressors) that bind to the DNA sequences (operators) within promoter regions in *C. glutamicum* have been classified on the basis of their regulatory hierarchy level into three groups, global, master and local (Schröder and Tauch, 2010). To elucidate the effects of environmental changes and the metabolic state of the cell on the promoter activity, the transcriptional regulator(s) involved should be identified and the respective DNA binding sites determined. The web-based analysis platform CoryneRegNet was established to handle experimental data related to transcriptional regulation in *C. glutamicum* (Baumbach *et al*., 2009; Pauling *et al*., 2012). Based on bioinformatic analyses, 159 *C. glutamicum* regulators and their regulatory interactions were suggested. According to the current data, it is apparent that a large number of *C. glutamicum* genes are regulated by TRs. Moreover, 158 genes were found to be regulated by two TRs, 46 genes by three TRs and 15 genes by four or five TRs (Schröder and Tauch, 2010). Many *C. glutamicum* promoter regions thus carry the binding sites for multiple TRs. In total, 452 DNA-binding motifs (September 2012) have been defined by experimental and bioinformatic approaches and the resulting data are available at CoryneRegNet. The temporal transcription pattern of a single promoter therefore result from the integration of the effects of several regulating factors ensuring a coherent response to environmental and metabolic changes. To identify TRs involved in the control of *C. glutamicum* promoters, a number of methods have been used.

Combining bioinformatic approaches and electrophoretic mobility shift assays enabled a GlxR (CRP-family) TR to be defined as a *C. glutamicum* global regulator controlling about 14% of the annotated *C. glutamicum* genes (Kohl *et al*., 2008; Kohl and Tauch, 2009). The use of chromatin immunoprecipitation combined with microarray analysis (the ChIP-chip technique) revealed 209 GlxR binding sites in the *C. glutamicum* genome, which is in very good agreement with the *in silico*-predicted GlxR binding sites. The activation of the expression of selected genes by GlxR was confirmed by promoter – reporter assays (Toyoda *et al*., 2011). The same approaches as those used to define the global and master regulators controlling large sets of *C. glutamicum* genes were applied to characterize the regulation of the expression of a single gene by the action of multiple regulators. Transcriptional control of the expression of the *rpf2* gene (encoding the *C. glutamicum* resuscitation promoting factor) by the RamA, RamB and GlxR regulators is an example of such a complex regulation (Jungwirth *et al*., 2008). The site-directed mutagenesis of operator sequences can determine the significance of individual nucleotides in the control of gene expression by the action of TRs. Such a detailed analysis determined, e.g. the mechanism by which the LldR TR controls the expression of the *C. glutamicum lldD* gene coding for lactate dehydrogenase (Georgi *et al*., 2008).

## Use of *C. glutamicum* promoters for modulation of gene expression

### Constitutive promoters

The use of native *C. glutamicum* promoters for optimizing gene expression began relatively recently. Strong constitutive *C. glutamicum* promoters were used for the construction of expression plasmid vectors as well as for replacing the native promoters of the selected genes in the *C*. *glutamicum* chromosome. The constitutive promoter of the *cspB* gene, coding for the main *C. glutamicum* secreted surface-layer protein PS2, was applied to the construction of the *C. glutamicum* – *E. coli* expression vector pCC (Tateno *et al*., 2007). This vector subsequently served as a basis for the construction of specialized vectors used for the secretion or cell surface display of the products of the cloned genes (Tateno *et al*., 2009). The strong constitutive promoter of the *gapA* gene (encoding glyceraldehyde 3-phosphate dehydrogenase), cloned in a multi-copy plasmid vector, was used to overexpress the *iolT1* and *iolT2* genes, which proved their role in glucose uptake (Ikeda *et al*., 2011). Overproduction of the *C. glutamicum* succinate exporter was achieved by overexpressing the *sucE1* gene from the strong constitutive promoter of the *C. glutamicum tuf* gene encoding the translational elongation factor EF-Tu (Fukui *et al*., 2011).

The native promoters of selected genes in the *C*. *glutamicum* chromosome were replaced with strong constitutive promoters to obtain stable and efficient plasmidless strains producing lysine (Becker *et al*., 2005; 2007; 2011; Neuner and Heinzle, 2011; Neuner *et al*., 2012), diaminopentane (Kind *et al*., 2010; 2011) or succinate (Litsanov *et al*., 2012). The promoters of *C. glutamicum sod* (superoxide dismutase) and *tuf* (translational elongation factor) genes were found to be suitable for these purposes.

Overexpression of the *fbp* (fructose 1,6-bisphosphatase) and *zwf* (glucose 6-phosphate dehydrogenase) genes from P*sod* and P*tuf* significantly increased l-lysine production on glucose, fructose and sucrose (Becker *et al*., 2005; 2007). The simultaneous overexpression of the chromosomal genes *pyc* (pyruvate carboxylase), *dapB* (dihydrodipicolinate reductase), *lysC* (aspartate kinase) and *tkt* (transketolase) from P*sod* and of the *fbp* gene from P*tuf* was found to redirect major carbon fluxes towards l-lysine hyperproduction (Becker *et al*., 2011). The insertion of P*sod* upstream of the *dld* (d-lactate dehydrogenase), *pyc* and *malE* (malic enzyme) genes within the *C. glutamicum* chromosome resulted in a *C. glutamicum* strain producing l-lysine during growth on lactate (Neuner and Heinzle, 2011). The overexpression of the genes *fbp* and *gapX* (glyceraldehyde 3-phosphate dehydrogenase) from P*sod*, in addition to that of the *dld*, *pyc* and *malE* genes, enabled the production of l-lysine by the resulting *C. glutamicum* strain on grass and corn silages (Neuner *et al*., 2012).

P*tuf* was also used to overexpress the *E. coli ldcC* (lysine decarboxylase) gene inserted into the *C. glutamicum* chromosome, which resulted in an increased production of diaminopentane (cadaverine) by engineered *C. glutamicum* cells (Kind *et al*., 2010). The diaminopentane production by this *C. glutamicum* strain was further improved by overexpressing the *cg2893* gene, coding for a permease, from P*sod* (Kind *et al*., 2011). The *C. glutamicum* strain overexpressing the mutated *pyc* gene as well as the *Mycobacterium vaccae fdh* (formate dehydrogenase) gene integrated into the *C. glutamicum* chromosome from P*tuf* was found to produce a high amount of succinate from glucose and formate under anaerobic conditions (Litsanov *et al*., 2012).

### Inducible promoters

Inducible promoters are convenient tools for controlled gene expression and are crucial elements of constructed plasmid expression vectors. The vast majority of the *C. glutamicum* plasmid expression vectors used contain heterologous inducible promoters. These promoters include the heat-induced *P*_R_*P*_L_ promoters of phage λ (Tsuchiya and Morinaga, 1988) and the inducible *E. coli* promoters P*lac*, P*tac* and P*trc*, induced by isopropyl-β-d-thiogalactopyranoside (IPTG). The *C. glutamicum*/*E. coli* shuttle expression plasmid vectors containing these promoters have been listed and described in detail in our previous review articles (Nešvera and Pátek, 2008; Pátek and Nešvera, 2012). The IPTG-induced promoters can be successfully used for the controlled overexpression of the *C. glutamicum* genes at the laboratory scale but their use on industral scale is very limited due to the high cost of the inducer. There is still a demand for alternative efficient and cheap inducers for biotechnological applications.

The arabinose-inducible expression system has been developed for large scale applications. This system is based on the functionality of the *E. coli* P*araBAD* promoter in *C. glutamicum* (Ben-Samoun *et al*., 1999) and the *E. coli* genes *araC* and *araE*, coding for a positive regulator and l-arabinose transporter respectively. The level of inducible gene expression from P*araBAD* can be modulated using a different l-arabinose concentration over a wide range (Zhang *et al*., 2012). Very recently, both heterologous and *C. glutamicum* promoters were used to construct a tightly controlled tetracycline-inducible expression system for corynebacteria. In the expression vector pCLTON1, the genes to be overexpressed are inserted downstream of the modified *B. subtilis* P*tet* promoter, which is tightly repressed in the absence of tetracycline by the TetR repressor. The gene coding for TetR, carried by the same plasmid, is expressed from the strong constitutive *C. glutamicum* P*gapA* promoter. (Lausberg *et al*., 2012).

Few instances of the use of native inducible *C. glutamicum* promoters for controlled gene overexpression have been reported so far. *C. glutamicum* promoters induced by acetate (Gerstmeir *et al*., 2003), gluconate (Letek *et al*., 2006; Okibe *et al*., 2010), maltose (Okibe *et al*., 2010) or propionate (Plassmeier *et al*., 2012a) have been described.

**Figure 5:**
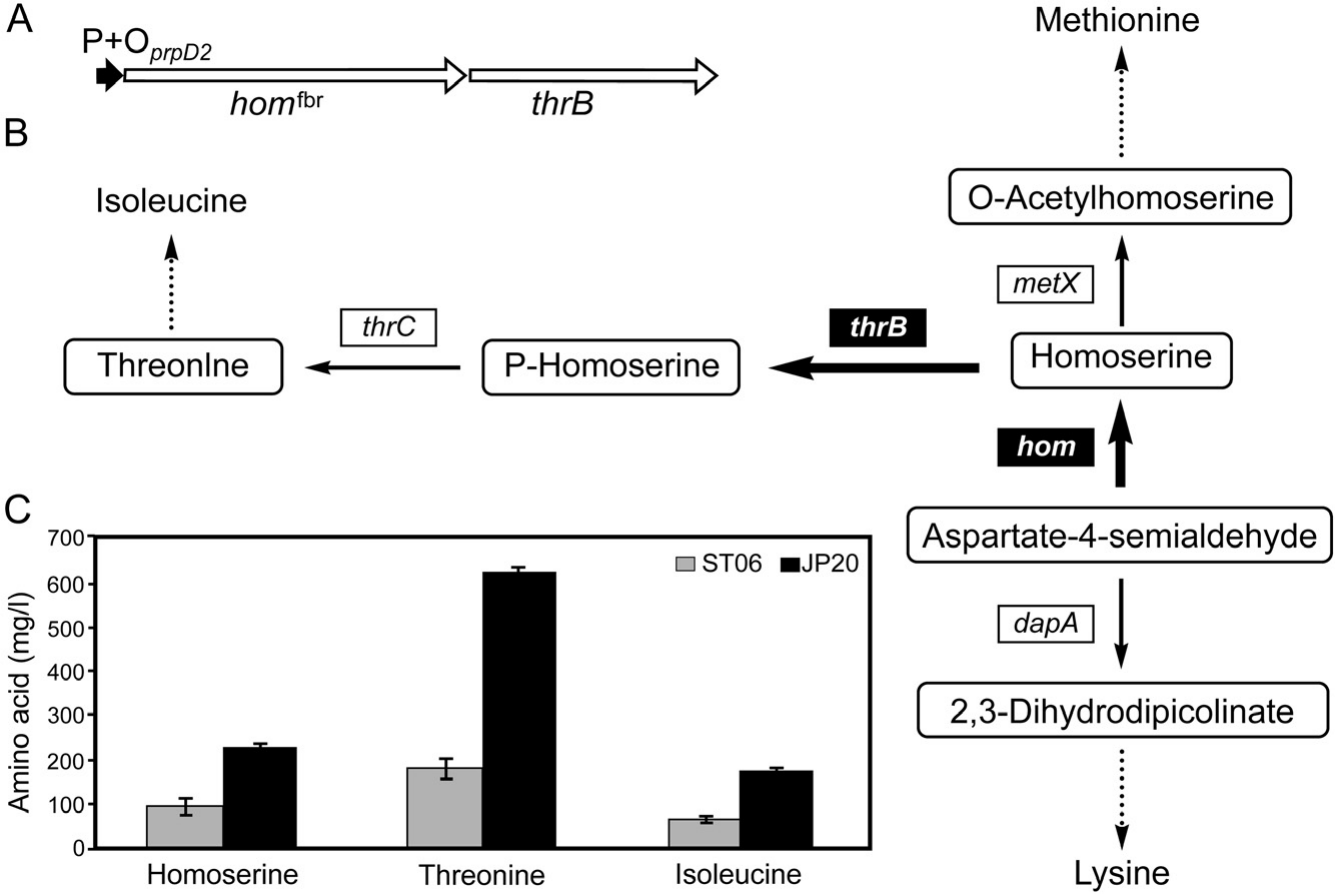
Redirection of metabolite flow in biosynthesis pathway of aspartate-derived amino acids using the propionate-induced P*prpD2* promoter.A. Insertion of the P*prpD2* promoter upstream of the *hom*-*thrB* operon in lysine-producing strain ST06, resulting in the strain JP20. *hom*^fbr^, the *hom* mutant gene coding for feedback-resistant homoserine dehydrogenase; P + O*prpD2*, promoter and operator of the *prpD2* gene.B. Metabolic pathway showing increased flux of metabolites from aspartate-4-semialdehyde to P-homoserine due to propionate-induced overexpression of *hom* and *thrB* genes coding for homoserine dehydrogenase and homoserine kinase, respectively, in the strain JP20.C. Concentrations of excreted amino acid after addition of propionate to both cultures of ST06 and JP20, indicating higher synthesis of homoserine, threonine and isoleucine in the strain JP20 (Plassmeier *et al*., 2012a).

The gluconate-inducible promoter P*git1* and the maltose-inducible promoter P*malE1* were applied in the controlled expression of the *xynA* gene (coding for xylanase) from *Clostridium cellulovorans* in *C. glutamicum* (*Okibe et al.,* 2010). The observed strong induction of the promoter of the *prpDBC2* operon, coding for the enzymes of the 2-methylcitrate cycle, by propionate in the presence of the PrpR activator (Plassmeier *et al*., 2012a) served as a basis for the construction of a novel propionate-inducible system (Fig. 5). This expression system seems to be very convenient for use in both laboratory studies and industrial-scale applications, as it uses a cheap inducer in very small amounts (1 mg l^−1^) and functions in minimal and complex growth media. In addition, since the inducer (propionate) is consumed by the cells, it offers a transcription that drops when the inducer is exhausted. The system was successfully applied to redirect fluxes towards threonine in a lysine-producing *C. glutamicum* strain by using the propionate-inducible expression of the *hom* and *thrB* genes that code for homoserine dehydrogenase (the branchpoint enzyme) and homoserine kinase (catalysing the first step in threonine biosynthesis) respectively (Fig. 5) (Plassmeier *et al*., 2012b).

**Figure 6:**
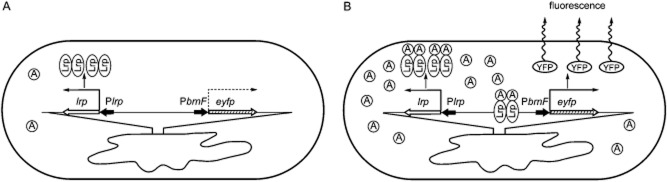
Bionsensor system for visualizing intracellular amino acid concentration within a single *C. glutamicum* cell using *eyfp* reporter gene.A. A sensor cell with low amino acid level exhibiting only background level of reporter gene expression.B. Induction of reporter gene expression, estimated as fluorescence increase, due to increased concentration of amino acid enabling its interaction with the Lrp activator (biosensor) and consequently resulting in the activation of the P*brnF* promoter. The thick arrows representing genes (empty or hatched) and promoters (short filled) indicate the direction of transcription. The thin bent arrows represent the mRNA transcripts (the dashed bent arrow indicates the low basal level of the transcript). Lrp, transcriptional regulator (sensor); YFP, yellow fluorescent protein (reporter); circled A; amino acid (methionine or a branched-chain amino acid) (adapted according to Mustafi *et al*., 2012).

The newly developed biosensors for the visualization of intracellular amino acid concentrations within single *C. glutamicum* cells are the most recent examples of entirely novel applications of inducible *C. glutamicum* promoters. In these systems, the increased concentration of an amino acid interacting with a positive TR (serving as a natural sensor) results in the activation of a promoter controlled by this regulator and consequently in the expression of a reporter gene whose product is easily quantifiable. Two such biosensor systems exploiting the reporter gene *eyfp*, coding for the enhanced yellow fluorescent protein which can be detected by fluorescence-activated cell sorting (FACS), have been developed for *C. glutamicum*. One system sensing the concentrations of branched amino acids and methionine by their interaction with the Lrp TR contains the promoter of the *brnEF* genes, coding for a two-component amino acid exporter, upstream of the *eyfp* reporter gene (Fig. 6) (Mustafi *et al*., 2012). The other system that senses the concentration of l-lysine by its interaction with the LysG activator contains the promoter of the *lysE* gene, coding for a basic amino acid exporter, upstream of the *eyfp* reporter gene (Binder *et al*., 2012).

### Modified promoters

In addition to the natural promoters, modified promoters constructed by site-directed mutagenesis of specific nucleotides within the promoter sequences have been used for the optimized expression of *C. glutamicum* genes. The *C. glutamicum* mutant promoters carrying alterations in the specific nucleotides within the −35 hexamers and the extended −10 regions are listed in Table 1. A set of promoters of various strengths was constructed by site-directed mutagenesis of the *C. glutamicum* promoter of the *dapA* gene coding for dihydrodipicolinate synthase (Vašicová *et al*., 1999). An analysis of the individual P*dapA* mutants revealed the significance of individual nucleotides within the −10 and −35 promoter sequences for promoter activity and contributed to defining the functional consensus sequences of *C. glutamicum* housekeeping promoters (Pátek and Nešvera, 2011). Some of the P*dapA* mutants were used for modulating gene expression aimed at the optimization of l-lysine (Pfefferle *et al*., 2003; van Ooyen *et al*., 2012) or putrescine production (Schneider *et al*., 2012). It was found that the introduction of a *dapA* gene copy with the strong mutant P*dapA*MC20 or P*dapA*MA16 promoter (Table 1) into the chromosome resulted in a significant increase in l-lysine yield in a *C. glutamicum* production strain (*Pfefferle et al.,* 2003). On the other hand, the weak mutant P*dapA*B6 promoter (Table 1) was used to initiate transcription of the *argF* (ornithine transcarbamoylase) gene, whose expression was further modified by changing the translation start codon and/or ribosome binding site. The strain producing the highest amount of putrescine so far was found among the *C. glutamicum* strains, harbouring the stably maintained plasmids with individual modifications and thus exhibiting different levels of ornithine transcarbamoylase (Schneider *et al*., 2012). The set of eight P*dapA* mutants (Table 1) was used (in addition to the wild-type P*dapA*) to achieve a gradual expression of the *C. glutamicum gltA* gene encoding citrate synthase. The obtained series of *C. glutamicum* strains with gradually decreasing citrate synthase activity was analysed at the transcriptome, metabolome and fluxome level and the l-lysine yield was found to be inversely proportional to the activity of citrate synthase. Using this approach, the *C. glutamicum* strain producing the highest amount of l-lysine on minimal medium with glucose so far was isolated (van Ooyen *et al*., 2012).

**Table 1:** Mutations in *C. glutamicum* promoters and their effect on promoter activity

Promoter	Gene product	−35	Extended −10	Up/Down effect of mutation	Reference
P*dapA*(WT)	Dihydrodipicolinate synthase	TAACCC	AGGTAACCTTG	–	(1)
P*dapA*MA16		TAACCC	AGGTA**TAA**TTG	Up	(1) (2) (3)
P*dapA*MC20		TAACCC	**T**GGTAACCTTG	Up	(1) (2)
P*dapA*A25		TAACCC	AGGTA**T**C**A**TTG	Up	(1) (3)
P*dapA*A14		TAACCC	AGGTA**T**CCTTG	Up	(1) (3)
P*dapA*A23		TAACCC	AGGTAAC**A**TTG	Up	(1) (3)
P*dapA*L1		TAACCC	AGGTA**GAA**TTG	Up	(1) (3)
P*dapA*C7		TAACCC	**TA**GTAACCTTG	Down	(1) (3)
P*dapA*B6		TAACCC	AGG**C**AACC**A**TG	Down	(1) (3) (4)
P*dapA*C5		TAACCC	**TT**GTAACCTTG	Down	(1) (3)
P*dccT*(WT)	Dicarboxylate transporter	CTACCA	CGTTAATATTC	–	(5)
P*dccT*FSM(SSM)		CTACCA	**T**GTTAATATTC	Up	(5)
P*dctA*(WT)	Dicarboxylate transporter	TTGCGT	TTTCATAATTT	–	(6)
P*dctA*MSM		TTGCGT	TTT**T**ATAATTT	Up	(6)
P*gdh*(WT)	Glutamate dehydrogenase	TGGTCA	TGCCATAATTG	–	(7)
P*gdh*2		TGGTCA	TGC**T**ATAATTG	Up	(8) (9)
P*gdh*3		T**T**G**A**CA	TGC**T**ATAATTG	Up	(8)
P*gdh*4		T**T**GTCA	TGC**T**ATAATTG	Up	(8)
P*gdh*7		T**T**G**C**CA	TGC**T**ATAATTG	Up	(8)
P*gdh*527_2		TGGTCA	TGCCATAA**A**TG	Down	(9)
P*gdh*527_3		TGGTCA	**CC**CCATAATTG	Down	(9)
P*gdh*527_4		TGGTCA	**CC**CCATAA**A**TG	Down	(9)
P*ilvD*(WT)	Dihydroxyacid dehydratase	GTGATA	AGCACTAGAGTGT	–	(10)
P*ilvD*M7		GTGATA	**T**G**TG**CTA**T**AGTGT	Up	(10)
P*ilvD*M14		GTGATA	AGCACT**GTG**GT**A**T	Up	(10)
P*ilvE*(WT)	Transaminase	GTGTAT	AGGTGTACCTTAA	–	(10)
P*ilvE*M6		GTGTAT	**T**G**TG**GTACC**A**TAA	Up	(10)
P*ilvE*M3		GTGTAT	AGGTG**CTC**CTTAA	Down	(11)
P*ilvA*(WT)	Threonine deaminase	TAGGTG	GATTACACTAG	–	(12)
P*ilvA*M1CG		TAGGTG	GAT**C**ACA**G**TAG	Down	(10) (13)
P*ilvA*M1CTG		TAGGTG	GAT**C**AC**TG**TAG	Down	(10)
P*leuA*(WT)	Isopropylmalate synthase	TACCCA	TTGTATGCTTC	–	(14)
P*leuA*M3A		TACCCA	TTGTATGC**A**TC	Down	(10)
P*leuA*M2TCG		TACCCA	TT**TC**A**G**GCTTC	Down	(10)
P*leuA*M2C		TACCCA	TTG**C**ATGCTTC	Down	(10)

References: (1) Vašicová and colleagues (1999); (2) Pfefferle and colleagues (2003); (3) van Ooyen and colleagues (2012); (4) Schneider and colleagues (2012); (5) Youn and colleagues (2008); (6) Youn and colleagues (2009); (7) Börmann and colleagues (1992); (8) Asakura and colleagues (2007); (9) Hänssler and colleagues (2009); (10) Holátko and colleagues (2009); (11) Hüser and colleagues (2005); (12) Pátek and colleagues (1996); (13) Hou and colleagues (2012); (14) Pátek and colleagues (2003b).

Stronger mutant derivatives of the promoter of the *gdh* gene, coding for glutamate dehydrogenase, were constructed (Table 1) with the aim of increasing l-glutamate production by the *odhA*-deficient *C. glutamicum* strain (lacking 2-oxo-glutarate dehydrogenase) (Asakura *et al*., 2007; Hänssler *et al*., 2009). Mutations within the −10 region enhanced glutamate dehydrogenase activity as much as 4.5-fold and the mutations in both the −10 region and −35 hexamer resulted in a further increase in the activity of this enzyme (sevenfold) (Asakura *et al*., 2007).

Mutagenesis of the native promoters of genes involved in the biosynthesis of valine, isoleucine and leucine was carried out within the chromosome with the aim of improving the l-valine production by *C. glutamicum* (Holátko *et al*., 2009). Up-mutations within the promoters of the *ilvD* (dihydroxyacid dehydratase) and *ilvE* (transaminase) genes (Table 1) were found to increase the activity of the respective enzymes involved in valine biosynthesis. On the other hand, down-mutations were constructed in the promoters of the *ilvA* (threonine deaminase) and *leuA* (isopropylmalate synthase) genes (Table 1), coding for enzymes which channel the flux of metabolites to the unwanted side-products isoleucine and leucine. Combining particular promoter mutations resulted in a plasmidless *C. glutamicum* strain exhibiting an enhanced production of l-valine (Holátko *et al*., 2009). A down-mutation in P*ilvE* (Table 1), resulting in a substantial decrease in ketoisovalerate flow to l-valine was used in the construction of a *C. glutamicum* pantothenate producer (Hüser *et al*., 2005).

It was found that novel metabolic capacities of *C. glutamicum* cells can be achieved by selecting spontaneous mutations within promoter regions. The ability of *C. glutamicum* cells to utilize succinate, fumarate or l-malate as the sole carbon source was observed when spontaneous mutations within the promoter sequences occurred and caused overexpression of the *C. glutamicum* genes *dccT* (Youn *et al*., 2008) and *dctA* (Youn *et al*., 2009), coding for dicarboxylate transporters. A spontaneous *C. glutamicum* mutant able to grow on glucosamine as a single carbon source was also recently isolated. The analysis of this mutant revealed that this newly acquired property was caused by a single mutation within the promoter of the *nagAB-scrB* operon. In contrast to the above-mentioned mutant promoters, this mutation is located outside the −10 and −35 sequences of the P1-*nagA* and P2-*nagA* promoters (Uhde *et al*., 2012).

## Conclusions and outlook

Most textbook examples of transcription initiation control mechanisms, which serve as paradigms for promoter regulation, such as the *lac* operon or *trp* operon in *E. coli*, are undoubtedly simplifications. An ever-expanding number of techniques applied to *C. glutamicum* are enabling us to analyse promoters in more detail and elaborate more precise models of promoter activity when subject to various environmental stimuli, nutritional conditions, stress situations and growth phases. Molecular methods provide data on the effects of TRs and other factors determining promoter activity under specific conditions. Using *in vitro* transcription, which mimics many features of *in vivo* transcription, the promoters can be classified on the basis of which σ factors recognize their core sequence. However, simplified working models must still be used to analyse particular regulatory functions. Since any promoter is a single cog in the cell machinery that forms a regulatory network, genome-wide technologies provide more complex information, which get us closer to understanding the cell on the level of systems biology. Transcriptomics and RNA sequencing enable the comprehensive detection and characterization of many promoters in parallel. In addition to the precise localization of the 5′-ends of mRNA, RNA-seq can provide quantitative information on promoter activity and transcript stability. In combination with proteomic, metabolomic and fluxomic data, our understanding of the cell at the system level is gradually improving. It is expected that the fusion of these global data sets will facilitate the construction of *C. glutamicum* strains that produce useful metabolites. Although a completely holistic approach to the description of the regulatory processes in a cell is still not practical, genome-scale metabolic flux determinations, metabolomic studies and the design of optimal metabolic pathways by *in silico* modelling have provided powerful tools for the optimization of producing strains (Becker and Wittmann, 2012; Vertes *et al*., 2012). At present, combining the data from system-level analyses with the findings obtained by the reductionist approaches to the description of regulatory mechanisms governing individual promoters seems to offer reliable information and tools for strain improvement in *C. glutamicum*.
